# Tree Invasions of Subarctic Shrublands Interact With Locally Augmented Snow and Functional Soil Depths: A Case Study in Denali National Park

**DOI:** 10.1002/ece3.71974

**Published:** 2025-08-19

**Authors:** Johanne O. Albrigtsen, Sarah E. Stehn, Carl Roland, Scott T. Allen

**Affiliations:** ^1^ Department of Natural Resources and Environmental Science University of Nevada Reno Reno Nevada USA; ^2^ Graduate Program of Hydrologic Sciences University of Nevada Reno Reno Nevada USA; ^3^ US National Park Service Denali National Park Denali Park Alaska USA; ^4^ US National Park Service Central Alaska Network Fairbanks Alaska USA

**Keywords:** global change, *Populus balsamifera*, stand structure, succession, tree encroachment, tussock tundra

## Abstract

Land‐cover changes and new ecosystem trajectories in Interior Alaska have altered the structure and function of landscapes, with regional warming trends altering carbon and water cycling. Notably, these changes include the increased distribution of tall woody vegetation, trees and shrubs, in landscapes that historically only supported low shrub vegetation cover. In Denali National Park, Alaska, this phenomenon has altered primary succession pathways towards tundra ecosystems with the establishment and expansion of balsam poplar ( *Populus balsamifera* ) trees. In this study, we examine how snow, soil, and vegetation processes interact within this altered successional pathway towards further landscape change following glacial recession. In a sequence of outflow terraces, we found that variations in snow depth, functional soil depth, leaf area index, overstory height, and understory height were all significantly correlated with each other, with those effects largely explained by the presence of poplar. Poplar‐dominated plots had deeper snowpacks, deeper functional soil depths, taller overstory and shrub heights, and greater LAI than in non‐poplar plots of the same landscape age. These findings suggest a feedback cycle where the establishment of taller vegetation (here, poplar) alters ecosystem processes in the following notable ways: taller vegetation is able to trap more snow by reducing wind exposure and limiting sublimation; this snow provides water through additional snowmelt and insulation, keeping soils warmer and lessening permafrost development, leading to deeper functional soil depths. This feedback demonstrates poplar's ability to modify the environment as an ecosystem engineer, engineering a trajectory away from the otherwise expected permafrost‐underlain tundra.

## Graphical abstract

In Denali National Park, Alaska, post‐glacial landscapes are undergoing a shift in successional pathways with the expansion of balsam poplar ( *Populus balsamifera* ), altering tundra ecosystem development. We found that poplar‐dominated plots had deeper snowpacks, warmer and deeper soils, and taller vegetation than shrublands, suggesting a feedback loop where poplar acts as an ecosystem engineer.

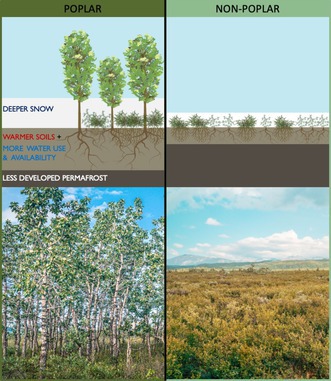

## 1 Introduction

Climatic warming is occurring four times faster in high latitudes relative to the rest of the globe (Rantanen et al. 2022). This warming is driving changes in vegetation composition, phenology, and productivity in boreal forests (Frost et al. 2023; Xi et al. 2024) and tundra (Lett and Dorrepaal 2018; Terskaia et al. 2020), including increasing the elevation and latitudinal limits of vegetation (Chen et al. 2011; Maher et al. 2021; Lett and Dorrepaal 2018). In the subarctic of interior Alaska, large‐scale vegetation changes, including migrating treelines and shrublines, are linked to increases in temperature (Miller et al. 2017; Maher et al. 2021; Dial et al. 2022). Thus, much of Alaska is at risk of warming‐induced landscape transformations, including complete biome shifts (Beck and Goetz 2011; Carlson et al. 2014). Although changes in air temperature alone are often insufficient to explain advancing vegetation changes (Rees et al. 2020; Payette 2007), indirect effects also occur, such as changing snow regimes that alter the water limitations that control plant distributions (Pauli et al. 2012). Together, the complex interplay between climate warming and ecosystem processes is driving vegetation change across northern landscapes.

Winter‐time ecosystem processes are particularly important in the subarctic, where landscapes often remain snow‐covered for over half the year, and permafrost dynamics and vegetation succession can be strongly coupled with temperature and snow regimes (e.g., Walker et al. 1993). Seasonally snow‐dominated landscapes exhibit heterogeneous snow distribution resulting from interactions among vegetation, topography, and wind (Gerber et al. 2017; Vionnet et al. 2021; Winstral et al. 2013). Vegetation influences snow accumulation and persistence on the landscape as well as snowpack properties over time (e.g., Barrere et al. 2018; Liston et al. 2002; Tennant et al. 2017; Sturm et al. 2001; Sturm, Douglas, et al. 2005). In northern latitudes, large‐statured vegetation can intercept wind‐blown snow, increasing snow depth and diminishing winter water losses from sublimation driven by wind (Pomeroy et al. 2006; Sweet et al. 2014; Sexstone et al. 2018; but also see Jost et al. 2007). Thus, changes in vegetation structure associated with regional/global climatic changes could also affect snow distribution, representing further modifications to microenvironments experienced by that vegetation.

Localized snow accumulation has diverse direct and indirect impacts on ecosystems in cold and warm seasons (Wipf and Rixen 2010; Niittynen and Luoto 2018). Snow regime affects water availability, soil and leaf temperature, surface microclimate, and timing and duration of the growing season (Amagai et al. 2018). Given its control over soil moisture, snowmelt impacts water and nutrient availability for vegetation in the growing season (Molotch et al. 2009). Snow cover is also a thermal insulator, with deeper snowpacks implying more insulation of soil and vegetation from cold winter air temperatures, reducing soil freezing and winter desiccation and wind abrasion of vegetation (Oakley et al. 1999; Sturm and Holmgren 1994). Even a snowpack of 25–30 cm depth has been shown to insulate the soil from daily air temperature fluctuations (Decker et al. 2003; Cleavitt et al. 2008; Iwata et al. 2010). Consequently, there is a relationship between snow depth and permafrost distribution (Zhang et al. 1997), a major control over vegetation, with greater snow facilitating higher soil temperature, greater seasonal thaw, and deepening permafrost depths (Slater et al. 2017; Myers‐Smith and Hik 2013). Evidence suggests that winter processes are contributing to vegetation conversions through a positive feedback that involves both the snow‐holding capacity of larger, woody vegetation and the insulating properties of snow (Sturm et al. 2001; Sturm, Schimel, et al. 2005; Euskirchen et al. 2016). Thus, increased snow depths can hypothetically lead to increased functional soil depths, which increase the volume of unfrozen soil available for water storage, nutrient cycling, and growing space for vegetation.

In Denali National Park, balsam poplar ( *Populus balsamifera* L., Salicaceae) trees are increasingly establishing at higher elevations than previously seen, in early‐ and mid‐successional landscapes that were on trajectory to become low shrub‐sedge tussock‐moss tundra underlain by shallow permafrost (Viereck 1966; Roland et al. 2016); this encroachment prompts questions of what other ecosystem changes are coming. Though balsam poplar is native to this region of the subarctic, its recent establishment occurs beyond its historical elevational range. Balsam poplar is a fast‐growing and generally short‐lived deciduous tree common in floodplain and upland sites throughout boreal North America (Burns and Honkala 1990). However, in our study area, poplar trees are newly present in diverse successional stages, creating a mosaic with patches of woodland dominating the short‐stature shrublands (Roland et al. 2016). It is unclear how this mosaic could affect overall successional trajectories and whether these patches will expand to completely replace the shrublands. It is important to understand these dynamics because extensive changes in vegetation and soils can imply changes in water, carbon, and nutrient cycling, with major downstream impacts on natural resources (as observed in high altitude forests: Peng et al. 2009; Dirnböck and Grabherr 2000; as observed across the arctic: Myers‐Smith et al. 2011).

This study examines the interplay of vegetation, snow, and permafrost, toward better understanding the ecosystem changes observed in Denali National Park. To test the impacts of expanding balsam poplar woodlands, we revisited the Muldrow outflow terraces (Viereck 1966; Roland et al. 2016) and measured vegetation structure (12 years since last measured), seasonal soil thaw depth, and snow depth distributions across several winter seasons. We compare traits of plots with and without poplars in a landscape that was devoid of poplar woodlands 70 years ago to investigate a hypothesized feedback cycle: poplars enhance snow trapping, provision water in early summer, and enhance the insulation of soils from cold air temperatures in winter, altering permafrost formation and thaw, together leading to more ideal and productive growing conditions for vegetation.

## 2 Background on the Muldrow Outflow Terraces

**FIGURE 1:**
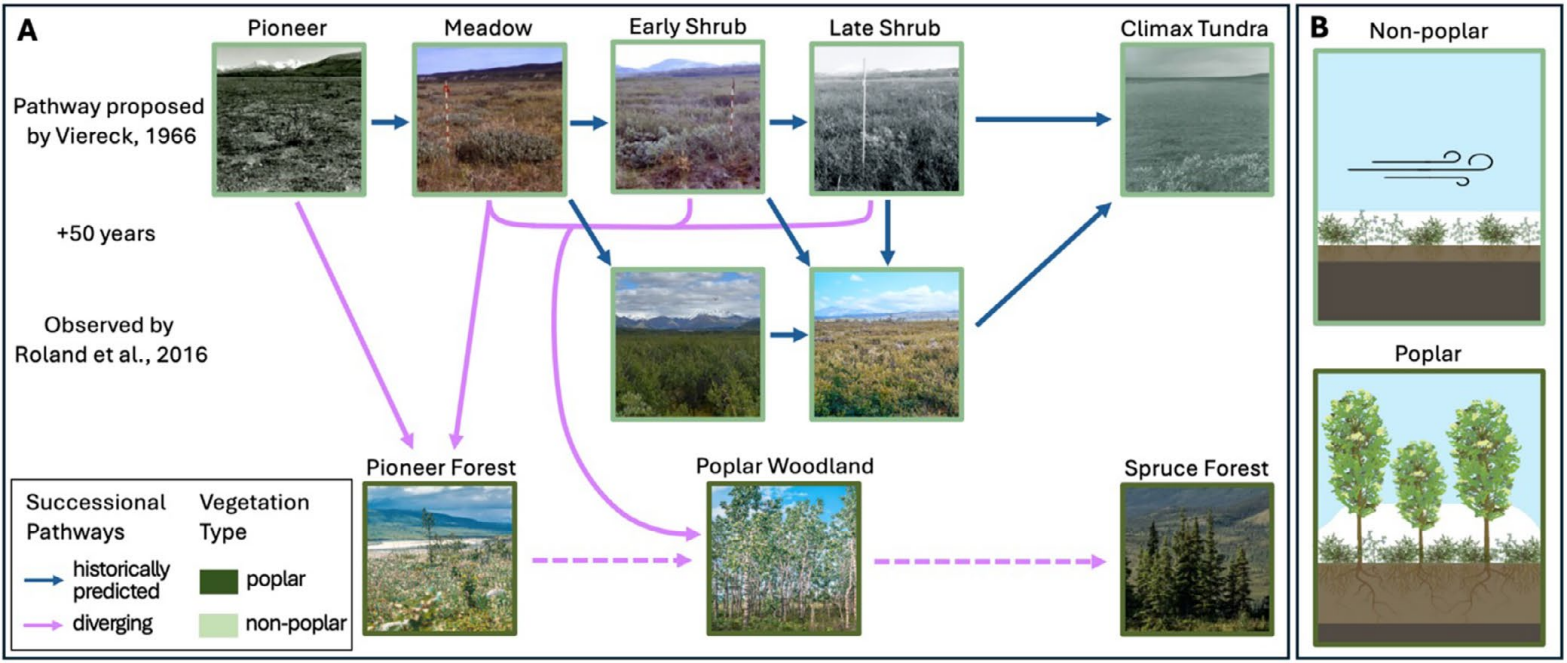
Overview of change across the Muldrow terrace chronosequence. (A) The top row represents the successional pathway proposed by Viereck 1966, which shows, from left to right, aging of the landscape involving succession through distinct vegetation types, eventually stabilizing as a climax tundra ecosystem. The second row represents a modified successional trajectory, proposed by Roland et al. 2016, based on observations that occurred 50 years later. Some locations within each terrace followed the predicted trajectories (blue arrows): Transitioning from meadow to early shrub stage, early shrub stage to late shrub stage, and the late shrub stage maintaining its characteristics. Other locations diverged from Viereck's predicted pathway: The pioneer and some of the meadow plots did not develop into the meadow stage and instead, with the establishment of poplar seedlings, were developing into a pioneer poplar forest; patches within the rest of the terraces (except Terrace 5) became dominated by mature poplar trees and are now poplar woodlands. Given that balsam poplar forest is generally a seral type that is succeeded by white spruce, it could be speculated that the poplar woodlands would eventually be replaced by a white spruce forest (new divergent future predictions, dashed pink arrows). (B) Currently, the most visually apparent variation within the chronosequence is not marked by transitions from one terrace (age) to the next, but by the presence and absence of poplar. This illustration shows the physical differences between the poplar‐dominated and non‐poplar landscapes explored by this paper: Aboveground vegetation structure, snow depth, and functional soil depth.

In 1958, a series of study plots was established on the outflow terraces of the Muldrow Glacier in Denali National Park (Denali) to study pathways of primary succession (Viereck 1966). Viereck (1966) used the outflow terraces as a space‐for‐time analysis of vegetation and soil development on a geomorphic chronosequence, i.e., a series of plots that share attributes but represent different ages since disturbance. Comprising four distinct outflow terraces (Terraces 1–4) and a fifth much older upland landscape (Terrace 5), their ages were estimated in 1958 to be 25–30 years for Terrace 1, 100 years for Terrace 2, 150–200 years for Terrace 3, 250–300 years for Terrace 4, and 5000–9000 years for Terrace 5 (Viereck 1966). Terraces 1–4 were classified into progressive stages of ecosystem development (Figure 1A), while Terrace 5 was used to represent a “climax” or peak stage of low shrub‐sedge, tussockmoss tundra (Viereck 1966). Viereck used the chronosequence represented by this landscape to infer and explain how vegetation and soil processes interact over time, providing a foundational model for understanding primary succession in post‐glacial, subarctic environments.

Viereck's study became a classic model of primary succession in the subalpine subarctic, describing vegetation changes and how they relate to changes in the physical environment. Soils freeze during the winter in Denali, but they can also experience substantial thawing in the summer, creating a deep functional soil layer. However, Viereck hypothesized that as moss and organic layers become thicker with successional progression, they insulate soils during the summer, and seasonal soil thaw becomes progressively slower, leading to permafrost layer development. Thus, the permafrost table is expected to rise closer to the surface as vegetation progresses from a meadow stage, through various shrub stages, to eventually stabilize as a low shrub‐sedge tussock‐moss tundra underlain by shallow permafrost (Figure 1A; Viereck 1966). Viereck's study presented a model of how vegetation succession not only transforms plant communities but also drives the formation and stabilization of permafrost in subalpine subarctic ecosystems.

This chronosequence was revisited 54 years later to assess how a changing climate may affect primary successional trajectories in Denali and the surrounding region (Roland et al. 2016). During the intervening years (1954–2012), the estimated mean monthly temperature during the growing season (May–August) significantly increased from a mean of ~9.5°C at mid‐century to a mean of ~11.5°C in 2009 (Roland et al. 2016). Roland et al. (2016) documented that over a half‐century interval, balsam poplar expanded widely across the study area (Figure 1A), marking a significant departure from Viereck's (1966) predicted successional trajectory. Where Viereck observed meadows and low shrubs, poplars had grown to form patches of woodland forest, representing a new ecosystem structure. Viereck had only documented a few balsam poplar seedlings in Terraces 1 and 2, and none in Terraces 3 and 4. The present poplar woodland communities have a taller canopy, reduced cover and depth of the moss mat, altered species composition with more forbs, and higher species richness of vascular plants than would be expected under the prior successional framework (Roland et al. 2016). However, this poplar proliferation is not universal, as other patches progressed relative to their landscape age as predicted by Viereck (Figure 1A, pink vs. blue arrows). Regardless, poplars were able to establish in early and middle stages of succession and persist, growing into mature trees, seemingly altering the trajectory of the landscape as a whole.

Viereck surmised that snow may play an important role in shaping this landscape and its vegetation. Roland et al. (2016) followed up on that suggestion and performed initial measurements of snow depths that indicated a correlation between poplar and snow worth investigating further. Almost 70 years after the original study, we expand upon the scientific legacy of this site to further investigate vegetation–snow–soil interactions as warming‐induced vegetation change continues (Figure 1B), with widespread poplar establishment apparently disrupting successional trajectories toward low shrub tundra.

## 3 Methods

### 3.1 Site Description

**FIGURE 2:**
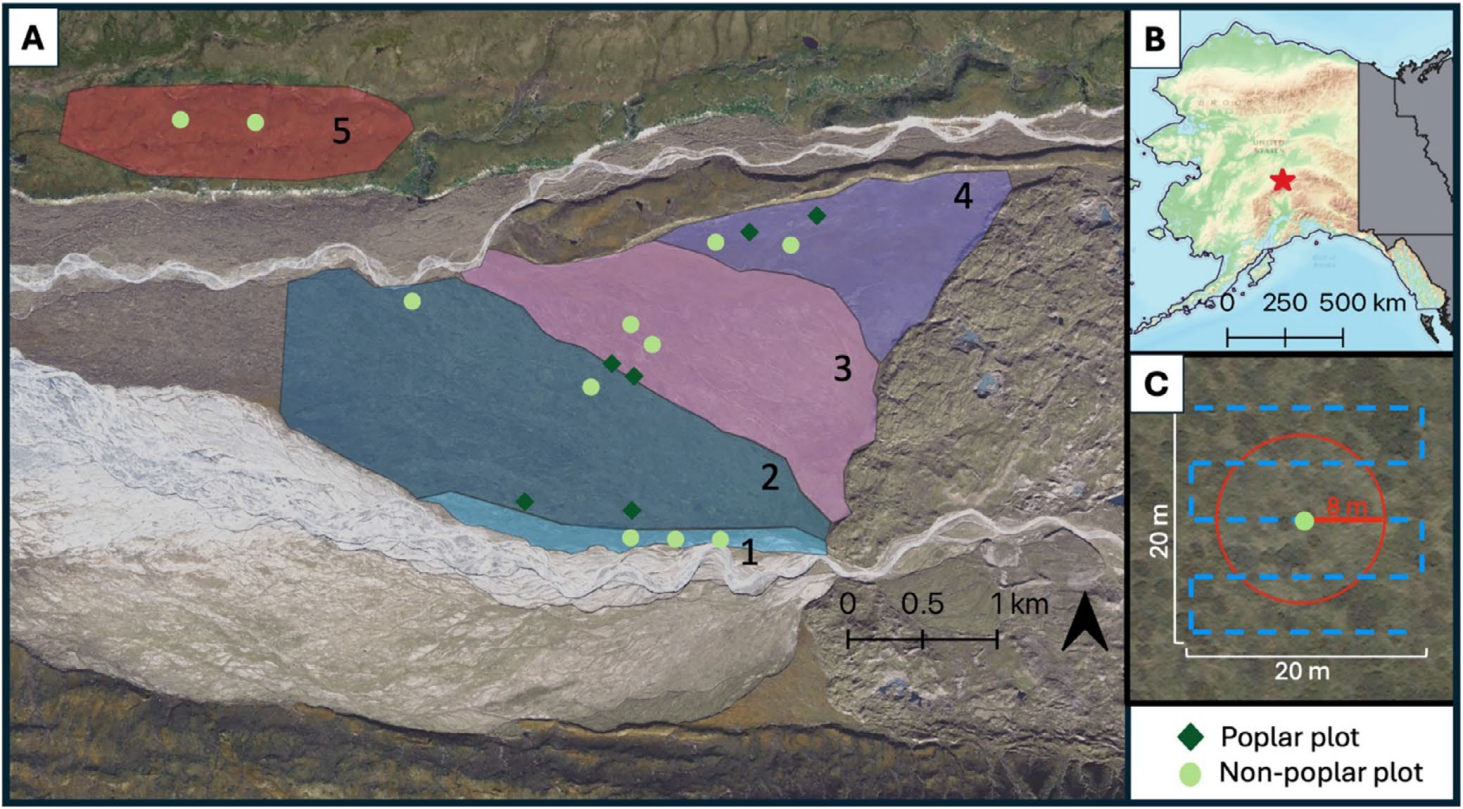
(A) Site map showing terrace boundaries (numbered 1–5, spatial boundaries distinguished by color) and plot locations. Contemporary ages (years) for terraces 1–4 are 95–100, 170, 220–270, 320–370, respectively, while terrace 5 can still be considered in the 5000–9000‐year age range. (B) Study area location (red star) within Alaska. (C) Plot‐level sampling designs for winter snow surveys (blue grid) and summer vegetation and soil surveys (red circle) relative to plot centroid.

The study area is situated on the northern slope of the Alaska Range (63°25′ N, 150°35′ W) at a mean elevation of 750 m (Figure 2A,B). The site is located on an outwash plain of coarse gravel between the McKinley and Thorofare Rivers, west of the Muldrow Glacier's terminal moraine (minimum stabilization age of 1300 years; Dortch et al. 2010). The study area has a continental, subalpine climate with long, cold, dry winters and short, warm summers. Denali receives a mean annual precipitation of 40.4 cm, of which 16.3 cm falls as snow (1990–2020 climate normals; Sousanes and Hill 2024). Mean temperatures in January and June are −16.5°C and 11.7°C, respectively (1991–2020 climate normals; United States, US Department of Agriculture 2025).

The study area consists of four large and relatively homogenous terrace landscapes in varying degrees of soil and vegetation development and one representative “climax” landscape. These terrace surfaces are distinct, separated by several‐meter breaks in elevation, creating a stepped‐terrace landscape. Terraces are described here as a chronosequence, with Terrace 1 adjacent to the McKinley River and subsequent terraces to the north towards the Thorofare River (Figure 2A). Given that ~70 years that have passed since the original estimated age designations, contemporary ages (years) for Terraces 1–4 are 95–100, 170, 220–270, and 320–370, respectively (Viereck 1966). Terrace 5 is an older upland landscape, 5000–9000 years old, north of the Thorofare River used to represent a “climax” or peak stage of succession (Viereck 1966). The terraces were also classified into stages of ecosystem development (Figure 1A). Terrace 1, the youngest surface, was classified as being in a “pioneer stage” with an early‐seral herb community growing on recently colonized alluvium. Following, Terrace 2 was classified as a “meadow stage” community, characterized by the expansion of herbaceous cover. Viereck suggested that the meadow stage transitions to an “early shrub stage” like Terrace 3, characterized by an open low shrub canopy of dominantly dwarf birch ( *Betula nana* ). Terrace 4 was classified as a “late shrub stage”, characterized by a deepening of the moss layer and a closing of the shrub canopy. It was hypothesized that the shrub ecosystem develops into the stable “climax” stage of low shrub‐sedge, tussock‐moss tundra, which characterized Terrace 5 where primary succession began millennia earlier (Viereck 1966).

### 3.2 Sampling Design

To assess continued vegetation change and potential cascading ecosystem impacts, we revisited a subset of plots established by Viereck (1966) and/or Roland et al. (2016). Balsam poplar has effectively colonized patches of the landscape across Terraces 1–4 and has established woodland forests in Terraces 2, 3, and 4. However, non‐poplar vegetation also still exists across the chronosequence, and thus some extant patches still resemble the predicted community compositions relative to their development stage. As in Roland et al. (2016), we selected plots representing end members of the range of vegetation composition in each successional terrace—plots were classified into two categories, “poplar” and “non‐poplar” (Figure 2A). “Poplar” plots (*n* = 2 per terrace in terraces 2–4) are dominated by balsam poplar ( *Populus balsamifera* ) woodland with relatively homogeneous overstory cover. “Non‐poplar” plots (*n* = 2 per terrace, terraces 1–5) are dominated by the predicted vegetation types for their respective successional stage, with shrub cover, when present, comprised of predominantly dwarf birch ( *Betula nana* ) and/or willow (*Salix* spp.) without the presence of mature balsam poplar trees.

Terraces 1 and 5 do not allow for the comparison of poplar versus non‐poplar plots; they do aid in understanding interactions between vegetation, snow, and soils. Terrace 1 is the youngest landscape and is also experiencing widespread establishment of balsam poplar, though it is mostly young. Terrace 5, the oldest landscape, represents the end member of the predicted successional pathway and allows for comparison with the non‐poplar plots in the younger terraces.

Selecting sites representing ends of the vegetation spectrum within each terrace allows for best assessing the relationship of vegetation with potential differences in other landscape features (i.e., snow depth and distribution, and functional soil depths).

### 3.3 Vegetation Characteristics

Vegetation data were collected over the course of 2 weeks in the summers of 2022 (July 4–16), 2023 (July 3–14), and 2024 (July 28–Aug 6). Data were collected within an 8‐m radius circular plot (200 m^2^) centered on each of the previously established plot center coordinates (Figure 2C, red circle). Vegetation height (for shrubs and trees) was measured in all plots in 2023 and 2024. Tree density and tree basal area were measured at all plots in 2024. Shrub height was measured as height from ground surface to tallest woody twig for 5 individuals in each plot. In poplar plots, tree heights (*n =* 5 per plot) were determined with a clinometer and measuring tape. Tree density and basal area were determined through measuring diameter at breast height (DBH, 1.37 m) of every tree and sapling (height ≥ 1.37 m) and every seedling (height < 1.37 m) was counted in each plot. To assess canopy structure, plot‐level leaf area index (LAI) was determined in 2022 using a LICOR LAI‐2200C Plant Canopy Analyzer (presented as a mean from *n* = 25 to 35 measurements per plot), using a clear‐sky correction and a 53° view angle.

### 3.4 Functional Soil Depth Measurements

Permafrost is ground that remains at or below 0°C for at least two consecutive years and is overlain by the “active layer”, the upper layer of soil subject to annual thawing and freezing in areas underlain by permafrost. Active layer thickness, or seasonal thaw depth, is the thickness of this seasonally thawing and freezing soil layer and is typically measured as the maximum depth of thaw at a certain timepoint. The study area spans recently deposited gravel alluvium through continuously developed permafrost soils. We measured functional soil depth as the top layer of soil which either never freezes or thaws seasonally (the active layer) and is accessible for plant use as belowground growing space. The functional soil depth was defined as depth to permafrost in plots underlain by permafrost and as the depth to regolith in plots without permafrost. We determined the functional soil depth through measuring the depth to refusal (to the nearest cm) with a 1.5‐m tile probe (a simple metal rod with a handle with cm increments engraved).

Functional soil depth was determined in each plot the first week of July in 2022 and 2023 and the first week of August in 2024 within the same 8‐m radius circular plot as vegetation characteristics (Figure 2C, red circle). While these are not the seasonal maxima, which would occur in early autumn in most years, this measurement allows for intercomparison because all plots were sampled within the same week each year. Functional soil depths have high spatial variation; as such, multiple measurements were taken in a systematic grid in each plot to produce a statistical plot‐level average (*n* = 15–20 measurements per plot). In areas underlain by permafrost, some uncertainty results from the variable thickness of the transition layer and mixture of ice and water at the permafrost table. It is estimated that a single probe measurement has an accuracy of ±3 cm (Schaefer et al. 2014).

### 3.5 Snow Depth Measurements

Snow depth was manually measured once per year in 2021, 2022, 2023, and 2024. Surveys were conducted in March to capture late‐season snow accumulation. Snow depth was measured using a MagnaProbe, an instrument for rapidly measuring snow depth and location by GPS (MagnaProbe, SnowHydro; Sturm and Holmgren 2018). Snow depth measurements were taken approximately every 3 m in a 20 m × 20 m grid centered on the GPS‐estimated plot center (Figure 2C, blue grid).

On average, 51 measurements were recorded per plot per year (mean *n* = 51, SE = 3). Given that GPS uncertainties can be several meters and plot center stakes were buried by snow in the winter, snow depth was sampled in a larger area that enveloped the plots used for vegetation measurements to ensure the whole plot was captured. This precludes matching individual point‐level snow measurements to point‐level vegetation measurements.

The closest SNOTEL (NRCS) site, Kantishna (station ID: 1072), is located ~25 km NW of the study site. The SNOTEL data were used as a reference, independent of the vegetation effects on site, to assess relative snow accumulation in our study years and compare them to average values. Whereas the vegetation metrics were not remeasured each year, it was imperative to measure the snow depths in each year to determine whether any differences among plots occurred consistently, or alternatively only in winters with less‐than‐typical snowfall or winters with more‐than‐typical snowfall. Climatic variability among our four sampling years provided a wide variety of inputs (described further in Section 4).

### 3.6 Statistical Methods

To evaluate differences between mean values of measured variables in poplar and non‐poplar plots, Student's *t*‐tests were used. These tests were performed for each variable and each terrace separately, comparing poplar plots versus non‐poplar plots within Terraces 2, 3, and 4. Prior to conducting the *t*‐tests, the assumptions of normality, independence, and homogeneity of variance were checked. Normality was assessed visually using histograms and Q–Q plots, and homogeneity of variance was evaluated using Levene's test, with a threshold significance level of *α* = 0.05 for all tests. All statistical analyses were implemented using R (version 4.4.2; R Core Team).

Pearson's (parametric) and Kendall's (non‐parametric) correlations were quantified to examine first‐order relationships among plot‐level means of these measured variables: snow depth, functional soil depth, vegetation height (overstory and understory), and leaf area index (LAI). For streamlining this analysis, only snowpack data from 2021 were used because the 2021 snowpack was most representative of typical conditions for the region. Functional soil depth measurements and LAI from 2022 were used, while vegetation heights from 2023 were used. These correlation coefficients (*r* for Pearson and *τ* for Kendall) were quantified in R (version 4.4.2) using the PerformanceAnalytics and GGally packages. A threshold of *α* = 0.05 was adopted for assessing statistical significance.

While the t‐tests and correlations are presented because of their simplicity, generalized linear mixed models (GLMMs) were also used to examine relationships among snow depth, soil depth, and vegetation characteristics across all terraces, accounting for the nested study design. These were implemented using the lme4 package (Bates et al. 2015).

To test the poplar effect on snow depth across snow years, snow depth was modeled as a function of poplar presence (“poplar”, a binary fixed effect) with random intercepts for plot nested within terrace and for year to account for repeated measures:

(1) $$\text{Snow depth}\;\sim \;\text{Poplar}+\left(1|\text{Terrace}/\text{Plot}\right)+\left(1|\text{Year}\right)$$

We also fit separate GLMMs for each response variable while accounting for the effects of other variables that could potentially cause variations in the response variable:

(2) $$\text{Snow depth}\;\text{\textasciitilde{}}\;\text{Overstory height}+\mathrm{LAI}+\text{Poplar}+\left(1|\text{Terrace}/\text{Plot}\right)$$

(3) $$\begin{array}{c} \text{Soil depth}\;\sim \;\text{Overstory height}+\mathrm{LAI}+\text{Snow depth}+\text{Poplar}\\ +\left(1|\text{Terrace}/\text{Plot}\right) \end{array}$$

(4) $$\mathrm{LAI}\;\sim \;\text{Soil depth}+\text{Snow depth}+\text{Poplar}+\left(1|\text{Terrace}/\text{Plot}\right)$$

(5) $$\text{Overstory height}\;\sim \text{ Soil}\;\text{depth}+\text{Snow depth}+\text{Poplar}+\left(1|\text{Terrace}/\text{Plot}\right)$$

Poplar presence was included as a binary fixed effect indicating presence or absence of a poplar canopy (i.e., distinguishing poplar from non‐poplar plots), while terrace and plot were included as nested random intercepts to account for variation among sampling locations. For these models, the same data were used as for the correlation analysis, including only snowpack data from 2021. Models were fit using maximum likelihood; homoscedasticity and normality were evaluated using residual plots. Variance inflation factors were calculated with the car package (Fox and Weisberg 2019) and found to be below 5, indicating no substantial multicollinearity among predictors.

## 4 Results

**FIGURE 3:**
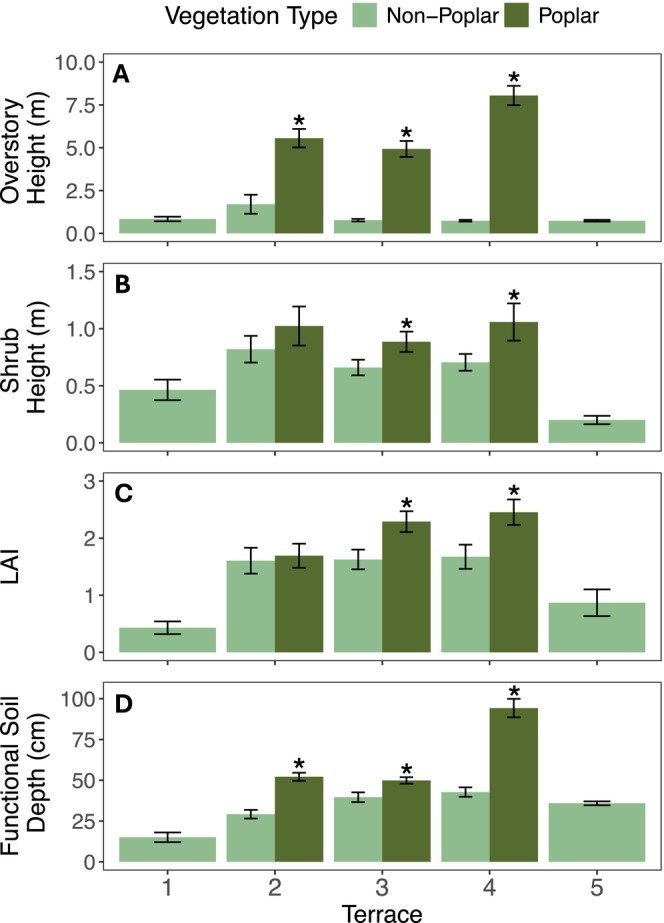
By terrace and vegetation‐type (poplar or non‐poplar) mean values for field measurements of (A) overstory height (m), (B) shrub height (m), (C) leaf area index (LAI, unitless), and (D) functional soil depth (cm). Error bars represent ±2 standard errors. *Denotes significant *p*‐values at *α* = 0.05 for *t*‐test statistics between vegetation types (poplar and non‐poplar) by terrace.

The influx of mature poplar trees introduced a new, much taller stratum of vegetation to the landscape, resulting in divergent vegetation structures (Figure 3A,B). This new poplar woodland vegetation structure (represented by the “poplar plots”) was observed in Terraces 2, 3, and 4, but was absent from Terraces 1 and 5. Vegetation height in the poplar plots averaged about five times that of the non‐poplar landscape (Table 1). In poplar plots, mean overstory heights in Terraces 2, 3, and 4 were 5.56 ± 0.27 m, 4.93 ± 0.23 m, and 8.05 ± 0.28 m (Table 1). By comparison, mean overstory heights (poplar saplings or shrubs) in the corresponding non‐poplar plots were significantly lower at 0.88 ± 0.04 m, 0.77 ± 0.04 m, and 0.74 ± 0.03 m (*p* < 0.05, Figure 3B). In poplar plots, the overstory was composed of balsam poplar with a shrub‐dominant understory, while in non‐poplar plots the shrubs mostly composed the overstory; all had relatively similar shrub heights (Table 1).

**TABLE 1:** Mean balsam poplar ( *Populus balsamifera* ) basal area for live trees (height ≥ 1.37 m), mean tree density, mean live seedling (height < 1.37 m) density, and mean shrub and tree heights (±1 SE) for all terraces and poplar (Y) and non‐poplar (N) plots measured in 2024; mean LAI (±1 SE) for each terrace and vegetation type measured in 2022.

Terrace	Poplar	Mean tree BA (m^2^/ha)	Mean tree density (stems/ha)	Mean live seedling density (stems/ha)	Mean shrub height (m)	Mean tree height (m)	Mean LAI
1	N	0	255	3989	0.50 ± 0.03	0.84 ± 0.07	0.43 ± 0.06
2	N	0.01	1464	4584	0.88 ± 0.04	1.70 ± 0.28	1.61 ± 0.11
	Y	4.72	3183	446	1.00 ± 0.05	5.56 ± 0.27	1.69 ± 0.11
3	N	NA	NA	0	0.77 ± 0.04	NA	1.63 ± 0.09
	Y	3.09	6303	4584	0.89 ± 0.04	4.93 ± 0.23	2.29 ± 0.09
4	N	NA	NA	64	0.74 ± 0.03	NA	1.67 ± 0.11
	Y	9.47	5411	1082	1.10 ± 0.06	8.05 ± 0.28	2.45 ± 0.11
5	N	NA	NA	0	0.21 ± 0.01	NA	0.87 ± 0.12

The youngest (Terrace 1) and oldest (Terrace 5) terraces had the lowest mean shrub heights at 0.5 m and 0.21 m, respectively (Table 1). Scattered poplar seedlings and some saplings were present in Terrace 1 (Table 1) while Terrace 5 remained dominated by tussock‐moss tundra with no evidence of poplar establishment.

The poplar plots containing tree‐sized individuals of balsam poplar contrasted in various ways with those where mature poplars were absent. In addition to the poplar plots having greater overstory heights, they showed relatively homogeneous poplar overstory cover and poplar basal areas between 3 and 10 m^2^/ha (Table 1). Mean tree density, measured as stems per hectare, was consistently highest in the poplar plots across all terraces, though the non‐poplar plots in Terrace 2 also showed notable tree density. In Terrace 1, mature trees and saplings (height ≥ 1.37 m) were sparse, resulting in a basal area of zero and a mean poplar density of only 255 stems/ha (Table 1). However, live poplar seedlings were prevalent within the shrub layer of all the poplar plots, as well as in non‐poplar plots in Terraces 1 and 2 (Table 1). LAI was significantly greater in the poplar plots compared to their corresponding non‐poplar plots in Terraces 3 and 4.

(*p* < 0.01; Figure 3C) but not Terrace 2 (*p* = 0.58; Figure 3C). The lowest mean LAI values occurred in Terraces 1 and 5 (0.43 and 0.87; Table 1).

Functional soil depth also varied across the chronosequence, with significant differences between poplar and non‐poplar plots (Figure 3D). In Terraces 2–4, functional soil depths were significantly deeper (*p* < 0.01) in poplar plots than in the non‐poplar plots in the same terrace (Figure 3D). Mean soil depths in poplar plots increased with terrace age: Terraces 2 and 3 averaged 52 ± 8 cm and 50 ± 6 cm, while Terrace 4 had the deepest mean of 94 ± 18 cm. Terrace 1, with its young alluvium deposits and rocky surface, had the shallowest soils, while Terrace 5 (underlain by permafrost) had a consistent mean depth of 35.9 ± 3 cm.

**FIGURE 4:**
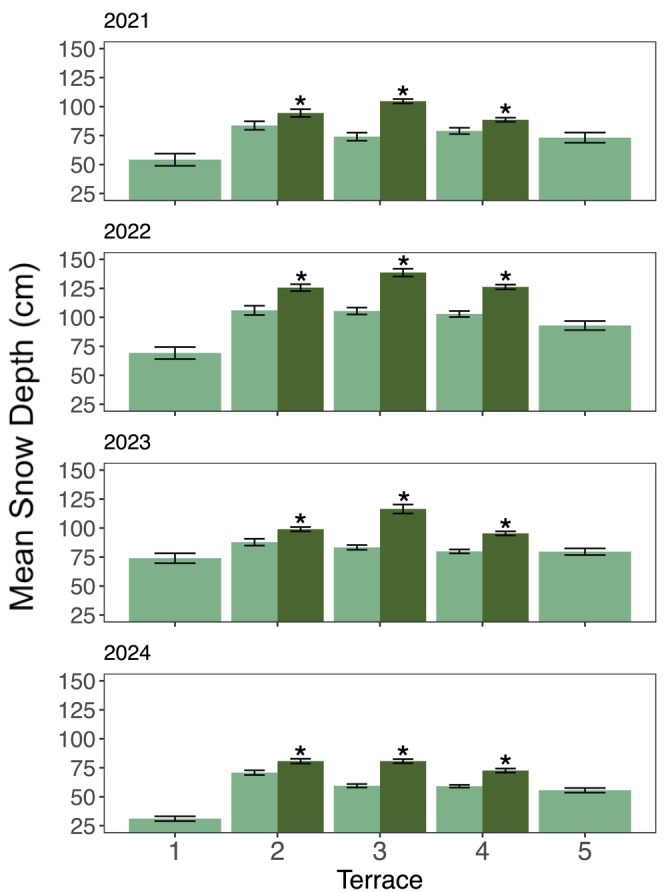
Mean snow depth (cm) with error bars representing ±2 standard errors, for years 2021–2024, across terraces and vegetation type (poplar, non‐poplar). *Denotes significant *p*‐values at *α* = 0.05 for *t*‐test statistics between vegetation types (poplar and non‐poplar) by terrace.

Snow depths were also consistently greater in poplar plots compared to those without, by terrace and across all years (Figure 4). On average, snowpacks in poplar plots were approximately 25% deeper than those in adjacent non‐poplar plots within the same terrace. This pattern was statistically significant (*p* < 0.01) within each terrace and study year (Figure 4). Moreover, GLMM analysis (Equation 1) showed a poplar effect of increasing snowpacks by an average of 18.2 ± 3.9 cm (±SE) compared to non‐poplar plots (*p* < 0.001), across years and Terraces 2–4. The shallowest snow depths were observed in Terrace 1, the youngest landscape, whereas Terrace 5, the late‐succession tundra, had depths similar to the non‐poplar plots in Terraces 3 and 4 across all years (Figure 4).

This pattern of poplar plots having deeper snow than their non‐poplar counterparts in each terrace (*p* < 0.01; Figure 4) occurred across years with notably differing winter precipitation inputs. Water year (defined as October 1 to September 30, named for the calendar year in which it ends; WY 2021–2024) snowpack depths ranged from greater than average (WY 2021 and WY 2022) to less than average (WY 2024). The total snow water equivalent (SWE, the depth of liquid water that would result from melting the entire snowpack at time of measurement) was 90.4 cm, 101.3 cm, 90.9 cm, and 62.5 cm in WY 2021–2024, respectively (United States, US Department of Agriculture, Natural Resource Conservation Service, National Water and Climate Center 2025). The difference in snow depth between poplar and non‐poplar plots was especially pronounced in WY 2022, which for both snow depth and precipitation was the most extreme year in the 99‐year weather record (Prugh et al. 2024; Figure 4). Snow depth patterns in WY 2023 mirrored WY 2021, with poplar plots again maintaining deeper snowpacks (*p* < 0.01; Figure 4). In WY 2024, the winter with the shallowest snowpack, the poplar effect persisted (*p* < 0.01; Figure 4), though with smaller differences between poplar and non‐poplar mean snow depths.

**FIGURE 5:**
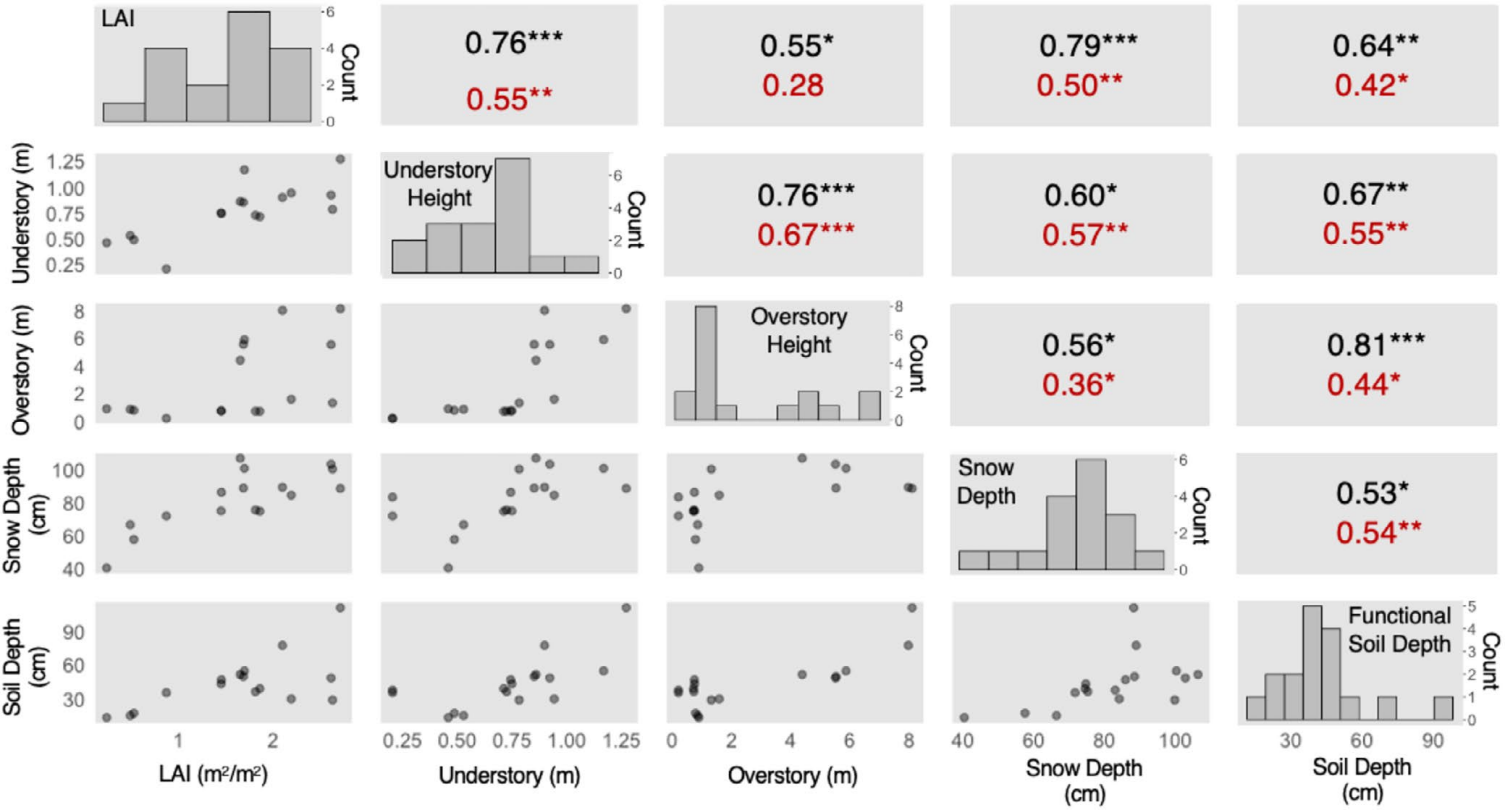
Scatter plots (bottom left corner), histograms (diagonal), and strength of correlations (top right corner) between key variables. The Pearson (black text) and Kendall (red text) correlation coefficients range from −1 to 1, with statistical significance represented by asterisks: *p < 0.05, **p < 0.01, and ***p < 0.001.

Beyond comparing poplar and non‐poplar plots by terrace, correlation analyses revealed significant relationships (*p* < 0.05) among overstory height, understory height, LAI, snow depth, and functional soil depth (Figure 5). Most of these positive correlations among variables were significant (*p* < 0.05) for both Pearson *r* values and Kendall *τ* (non‐parametric) values, implying that the vegetation structure and functional soil depths all increased together. GLMMs Equations ((2), (3), (4), (5)) were used to better understand those correlations and the potential poplar effect. After accounting for potential effects of overstory height and LAI on snow depth (*p* > 0.05; Table 2), a significant poplar effect on snow depth of 14.8 cm was observed (*p* = 0.008). Similarly, poplar plots had significantly deeper functional soil layers, averaging 21.3 cm deeper than non‐poplar plots (*p = 0.03*), with no significant effect of snow depth itself on active layer thickness once poplar presence was included (*p* = 0.80; Table 2). For vegetation structure, deeper functional soil layers were associated with a slight but significant increase in LAI (*p = 0.02*), independent of the poplar effect LAI (*p* = 0.56; Table 2). Alternatively, taller vegetation was associated with poplars (*p* < 0.001), and accounting for that poplar effect in this GLMM nullified the effects of snow accumulation or soil thaw depth (Table 2) that were apparent through correlation analysis (Figure 5).

**TABLE 2:** Summary of coefficients (±SE) from generalized linear mixed models (GLMMs) examining snow depth, functional soil depth, leaf area index (LAI), and overstory height.

Predictor	Models			
	Snow depth, model Equation (2)	Functional soil depth, model Equation (3)	LAI, model Equation (4)	Overstory height, model Equation (5)
(Intercept)	68.93 (3.14)*	37.02 (12.74)*	0.00 (0.68)	0.01 (0.97)
Poplar (Y/N)	14.88 (4.95)*	21.36 (8.79)*	−0.19 (0.31)	4.78 (0.56)*
Overstory height	0.36 (0.47)	1.21 (0.81)	NA	NA
LAI	0.37 (0.45)	0.81 (0.76)	NA	NA
Snow depth	NA	−0.03 (0.13)	0.02 (0.01)	0.01 (0.01)
Functional soil depth	NA	NA	0.01 (0.01)*	0.01 (0.01)

* *p* < 0.05.

## 5 Discussion

### 5.1 Hypothesized Feedback Between Vegetation Structure and Growing Conditions

We found that the substantial influx of balsam poplar into parts of this landscape has altered the physical structure of the vegetation mosaic and thereby changed patterns of snow accumulation, soil thaw depths, and vegetation structure (Table 2). We interpret these relationships as a result of a hypothesized “poplar effect” on the feedback among these attributes: (a) taller structure of poplar leads to deeper snowpacks than non‐poplar areas; (b) deeper snowpacks insulate soils in the winter, leading to warmer, less‐frozen soils; (c) improved growing conditions for trees further favor trees. In such a positive feedback cycle, a change in one variable (e.g., increased snow depth) could potentially cascade through the system, thereby magnifying the changes associated with climatic warming. While the cause–effect directionality of these relationships is unclear, our findings and others may indicate the mechanisms leading to these vegetation‐community variations despite all succeeding from alluvium (170–370 years ago for Terraces 2–4) and, more recently, from a treeless landscape (70 years ago; Viereck 1966).

We attribute the significantly higher snow depths in plots with poplar, and the general variation (e.g., from 30 to 150 cm over relatively short distances) to reflect interactions of snow and wind with vegetation heights across the mosaic of poplar woodland and dwarf‐birch‐dominated shrubland. This effect was pronounced given the minimal confounding features in this landscape with mild and consistent topography, spanning a major glacial moraine, which results in relatively uniform wind speed and direction across the sloped plain (personal observation across four multi‐week sampling campaigns). The robust influence of poplars on snow retention was demonstrated through consistently increasing snow depths in each study year, despite interannual weather conditions including below‐average to record‐breaking snow depths. This effect could occur by multiple potential mechanisms. Erosion is a means by which wind can affect the spatial distribution of snow; typically, more eolian erosion of snowpacks is seen in areas with shorter vegetation, such as shrubs, with that snow then depositing within the taller vegetation (Essery and Pomeroy 2004). Relatively taller vegetation can act like a snow fence, increasing aerodynamic roughness and boundary‐layer friction (i.e., relative to the lower shrub cover) and enhancing snow deposition, as has been observed in the alpine krummholz zone (Daly 1984) and in arctic tundra (Sturm et al. 2001). In addition to modifying the redistribution of the snowpack, the interception of wind‐blown snow (e.g., by large‐statured vegetation such as in this case balsam poplar) can reduce sublimation (Dickerson‐Lange et al. 2021); that is to say, poplars could yield net increases in snow depths across the landscape mosaic, rather than affecting redistribution. In other landscapes, increases in the snow‐holding capacity of a landscape within and downwind of tall vegetation patches have been observed, resulting in net gains in snowpack (Daly 1984; Sturm et al. 2001). In other cases, it has been observed that taller overstory vegetation and greater LAI can decrease snow accumulation on the ground due to canopy interception and subsequent melt or sublimation (Hedstrom and Pomeroy 1998; Storck et al. 2002; Musselman et al. 2008). Given our results, it seems that the dynamics of wind redistribution outweighed any role of interception loss in these poplar forests. Nonetheless, further elucidating the mechanisms by which poplars increase snow depths is an important next step because implications differ if snow gains result from (a) reducing wind‐driven sublimation, versus (b) deposition of snow scoured from lower‐stature vegetation; the latter is a product of the mosaic, and any such gains would subside if the entire landscape converts to poplar forest.

Winter snowpacks act to insulate the soil in winter, separating the soil from cold, subarctic airmasses and radiative cooling (e.g., Oakley et al. 1999; de Pablo et al. 2016), with differences of just 30 cm in snow depth capable of altering ground temperatures by as much as 5°C (Jorgenson et al. 2010; Pattison and Welker 2014). In deep snowpacks, soils maintain higher temperatures at the surface, causing seasonal thaw and permafrost depths to occur deeper within the soil profile, if permafrost occurs at all (Zhang 2005). Since permafrost formation and stability are influenced by aboveground temperatures, which are modulated by snowpack depth, a long‐term relationship between snow depth and permafrost distribution is expected (Shur and Jorgenson 2007). We found that snow depth and functional soil depths are positively correlated (Figure 5) and with 21‐cm deeper functional soil depths were associated with poplar stands (Table 2). However, snow depth alone did not significantly explain active layer thickness when poplar presence was also included in the model (Table 2). This finding suggests that the deeper snow and thicker soil layers are not simply an artifact of taller canopies, as seen elsewhere (Atchley et al. 2016; Wilson et al. 2020), but are instead driven by the structural and ecological role of poplar stands themselves.

In addition to poplar effects on functional soil depth (i.e., as influenced by relative thaw), poplars likely have other effects on permafrost development. Like snow, vegetation also insulates the soil, but whereas snowpacks insulate soils from cold winter air, vegetation, especially moss, shades the soil from solar radiation, lessening heating of soils in summer and promoting permafrost development. This is especially relevant because our study site falls within the discontinuous permafrost zone of the subarctic, where climate alone is insufficient to cause permafrost formation (Shur and Jorgenson 2007); here, “ecosystem‐driven” permafrost forms during late‐successional stages of ecosystem development (Shur and Jorgenson 2007; Viereck 1970), influenced by thick moss and organic layers (Viereck 1970; Van Cleve et al. 1983). Moss and humus layers insulate soils in the summer, keeping soil temperatures low (Harden et al. 2006; Romanovsky et al. 2008) and suitable for the development and rise of the permafrost table. Terrace 5, the climax tundra site that showed shallow functional soil depths (Figure 3D) and no evidence of poplar establishment, was previously noted to have a characteristic thick organic layer and fully developed shallow permafrost table (Roland et al. 2016). The non‐poplar plots in Terraces 3 and 4 had similar functional soil depths as Terrace 5 and can be seen as ecosystems in transition; at these sites, we directly observed ice at the bottom of soil‐core holes. Roland et al. (2016) also previously noted that the poplar plots differed by lacking some moss layers—the establishment of deciduous trees can impede moss development through increased leaf litter accumulation, shading, and chemical inhibition (Startsev et al. 2008)—potentially further contributing to the poplar effect on deepening functional soil depths and potentially precluding ecosystem‐driven permafrost formation (as modeled by Tutton and Way 2021; Way and Lapalme 2021).

Completing the hypothesized feedback cycle, the inferred poplar effects on warming soils and deepening thaw horizons may promote more productive and taller‐statured vegetation as compared to areas with thinner, colder soil profiles. Such improved growing conditions associated with soil warming have been shown elsewhere in Denali (Roland et al. 2012) and across interior Alaska (Roland et al. 2019). For example, annual growth in *Picea* spp. trees in interior Alaska is slower in permafrost soils than in deeply thawed soils (Nicklen et al. 2021), as is likely the case for many species, including poplars. In addition to warming increasing the physical belowground growing space, deeper functional soil layers have been found to be positively correlated with nitrogen availability in permafrost regions (Salmon et al. 2016; Hewitt et al. 2020). Others have specifically noted that balsam poplar is associated with increased forest floor nitrogen flux, although not necessarily accumulating in soils because it may be more readily taken up by the poplars themselves (Van Cleve et al. 1980). Poplars have been found to enhance the bioavailability of trapped soil organic matter (Goulden et al. 1998) and soil organic matter mineralization through improved soil drainage and oxygen availability (Hobbie et al. 2000). Others have also found poplars to increase inhibitory secondary compounds, such as tannins, that inhibit soil microbial activity and nitrogen mineralization in floodplain settings (Fierer et al. 2001), but it is unclear how those processes would apply in our sloped subalpine study site. Greater functional soil depth also means greater potential for water storage, complemented by the increased snow depth implying greater recharge by snowmelt, potentially extending the effects of snowmelt‐driven recharge on summer soil moisture (Maurer and Bowling 2014); this could directly benefit balsam poplars, given their physiological capability of high water use (Pointeau and Guy 2014). While root‐zone saturation can also limit trees by inducing hypoxic conditions, balsam poplar is seemingly quite resistant to such stressors (Landhäusser et al. 2003), as they can draw water from near the water table and the capillary zone above it. The fact that poplars, including balsam poplar, can be highly productive (Pointeau and Guy 2014), as well as the observed higher leaf area in poplar plots, suggests that the trees are making use of the enhanced growing conditions.

The changing growing conditions that likely benefit poplars (and seemingly also associated shrubs and other trees) may have differential beneficial or inhibitory effects on other species. Most prominently, the poplar effect seemingly shifts the system away from the expected progression toward low shrub‐sedge, tussock‐moss tundra (i.e., Terrace 5), in which permafrost and moss accumulation promote hypoxic, peat‐accumulating environments (Shur and Jorgenson 1998). Instead, the deeper functional soil depths in poplar plots (Figure 3D) should enhance drainage and decrease the likelihood or rate of peat accumulation (Sousa et al. 2021). Another effect of the poplars is extending the snowpack duration, with higher LAI (Figure 3C) increasing shading of the snowpack in addition to the greater snow inputs; readers should note that this effect was not measured because complete melt lagged our winter snow surveys. In terms of net impacts on snowmelt, there can be a tradeoff between vegetation shading and the lower albedo of vegetation masking the high albedo of snow (Sturm, Douglas, et al. 2005). In this system, however, snowmelt is dominated by short‐wave radiation rather than substantial melt caused by long‐wave radiation from the lower albedo of the poplar. As shown, snow depths were consistently greater under poplar compared to adjacent low‐stature tundra, suggesting that the snow‐trapping effect of the taller canopy, which reduces wind scour and sublimation, more than offsets any increased melt due to radiation absorption. Increased snow depths and durations can protect understory vegetation from winter desiccation and wind abrasion (Maher et al. 2019) and support the survival of species adapted to shaded environments with shorter growing seasons (Christiansen et al. 2018; Myers‐Smith et al. 2019). We observed that poplar plots in the mid‐successional terraces also had taller shrub layers than non‐poplar plots in the same terraces (Figure 3B), potentially reflecting poplar facilitation of understory plants; it was previously noted that the community composition of the shrub layer in poplar stands was considerably different than in non‐poplar plant communities (Roland et al. 2016). It has also been described that poplar can facilitate white spruce trees in lower‐elevation zones, importantly sheltering white spruce seedlings (discussed further in Section 5.3; Walker and Chapin 1986; Walker et al. 1986).

### 5.2 Trajectories of Change

A comparison of vegetation demography data from this study with 2012 measurements at the same plots (Table 3) highlights that shifts in the plant community are ongoing. Over that 12‐year interval, height growth and live basal area mostly continued to progress, but perhaps most notable was the increase in seedling establishment, particularly in the younger terraces (Table 3). In the early successional landscapes (Terraces 1 and 2), seedling density increased in non‐poplar plots, where poplar seedlings can continue establishing new patches of poplar woodlands. These changes likely reflect initial establishment of poplar by long‐range dispersal of small windborne seeds, enabling colonization of freshly exposed mineral soils and allowing for establishment early in primary succession (Zasada and Phipps 1990). In other plots, seedling densities decreased, suggesting that mid‐successional landscapes remain resistant to further poplar colonization, at least under current climatic norms (Table 1; it warrants noting that the non‐poplar percent change in seedling density in Terrace 3 is likely a sampling artifact rather than a true indication of change, see footnote in Table 3). Nonetheless, further poplar expansion can occur asexually, by stolons, stump sprouts, stem sprouts, and buried branches where edaphic conditions are favorable (Haeussler and Coates 1986; Krasny et al. 1988). We hypothesize that deeper unfrozen soils may support poplar's asexual reproduction strategy, allowing for rhizome‐driven colonization while translocating carbon deeper underground during winter (Comtois et al. 1986). Thus, our findings suggest that the poplar woodlands are increasingly initiating and maturing in a mosaic pattern, demonstrating the heterogeneous and dynamic nature of this ecological transition.

**TABLE 3:** Direct comparison between vegetation metrics measured in 2012 (Roland et al. 2016) and 2024 (data presented in Table 1).

Terrace	Poplar	2012				% change from 2012 to 2024			
		Live tree BA (m^2^/ha)	Seedling density (stems/ha)	Mean tree height (m)	Mean shrub height (m)	Live tree BA (m^2^/ha)	Seedling density (stems/ha)	Mean tree height (m)	Mean shrub height (m)
1	N	0	3506	0.5	0.45	0%	+14%	+68%	+11%
2	N	0	2785	0.9	0.5	+1%	+65%	+89%	+76%
	Y	3.29	746	5	0.8	+43%	−40%	+11%	+25%
3	N^a^	0	348	NA	0.6	0%	−100%^a^, ^b^	NA^a^	+28%
	Y	2.64	4849	4.25	0.65	+17%	−5%	+16%	+37%
4	N	0	75	NA	0.55	0%	−14%	NA	+35%
	Y	16.17	497	8	1.15	−41%	+118%	1%	−4%

^a^ Only one of the Terrace‐3 non‐poplar plots was also measured in 2012, thereby decreasing sample size and increasing uncertainty in all of the values.

^b^ Due to GPS uncertainty and the absence of permanent markers of plot centers, there may be small mismatches in the assumed plot center of each 8‐m radius plot; fieldnotes from 2022 to 2024 documented the presence of numerous poplar seedlings and saplings just outside the sampling plot boundaries, leading us to believe that this 100% is likely a sampling artifact due to GPS uncertainty.

Deviations from predicted successional pathways have been observed across the subarctic, and the rates of those changes have been quantified (Roland et al. 2016; Hollingsworth et al. 2010; Cutler 2011; Frost et al. 2023). In many cases, climate‐driven shifts in environmental conditions are altering underlying ecological processes, leading to departures from expected pathways and the emergence of unexpected successional trajectories (Anyomi et al. 2022; Williams et al. 2021; Walker and Wardle 2014). Aided by changing climatic conditions, the expansion of poplar is an example of this phenomenon: it alters typical primary successional patterns in the subalpine region of the subarctic, where shrubland vegetation has historically promoted permafrost formation, which in turn influences tundra vegetation composition (Viereck 1966; Shur and Jorgenson 2007).

Poplar's capacity for wind‐mediated long‐distance seed dispersal enables it to colonize new areas, and as such, as the climate warms, poplar is predicted to become more dominant north of the latitudinal tree line (e.g., Landhäusser and Wein 1993). Recent studies have documented the presence of extralimital stands of balsam poplar beyond the latitudinal treeline in Alaska (Breen 2014) and the Western Canadian Mainland Arctic (Saarela et al. 2012). While this northward expansion of tree species lines has been well documented, the elevational expansion of poplar also warrants further exploration.

At our site, poplar is pushing its local elevational distribution (see Roland et al. 2012). Poplar seedling recruitment typically requires a moist, bare, fine‐grained mineral seedbed (Zasada and Phipps 1990), significantly narrowing the scope and scale of potential poplar influx under current landscape conditions, as reflected by the absence of poplar in the climax tundra plots. However, the spatially widespread and accelerating retreat of glacial ice in Alaska (Roberts‐Pierel et al. 2022; Loso et al. 2014) is exposing vast areas of new mineral substrates, potentially relaxing this constraint. Such deglaciations could promote a “hopscotching” pattern of establishment—from existing stands near ice margins to freshly exposed glacial sediments—enabling rapid but patchy colonization of the post‐glacial landscape. Further, balsam poplar can facilitate white spruce establishment via shading, protection from winter climate, and associated moisture inputs (Walker and Chapin 1986; Walker et al. 1986). This relationship means that poplar expansion could pave the way for a transition towards spruce‐dominated forests, which would further shift these landscapes away from tundra and may intensify fire regimes (Johnstone et al. 2010; Abrahamson 2014; Johnstone and Chapin III 2006; Kasischke et al. 2010). In the rapidly changing arctic and subarctic, understanding these trajectories and their implications can help predict and manage the ecological impacts of ongoing climate change and disturbance (Post et al. 2009).

### 5.3 Implications of Co‐Occurring Shifts in Vegetation, Soil, and Snow Traits

Our findings suggest that balsam poplar occurrence in this ecosystem is associated with a role as an ecosystem engineer, modifying aboveground ecosystem structure, snow patterns, and soils. The impacts of ecosystem engineering by a single species can extend far beyond initial habitat modification, generating feedback loops that further reinforce the engineer's presence and influence (Gutiérrez and Jones 2008; Sanders and Frago 2024). Our findings prompt hypotheses of the poplar effect on water and carbon cycling.

The observed ecosystem changes and general expansion of poplar woodlands, e.g., by elevational and latitudinal treeline shifts, could have major effects on regional water cycling. On one hand, taller vegetation structure can decrease sublimation (Section 5.1), meaning greater snowmelt and inputs to soils. On the other hand, deepening soils increase soil‐water storage capacity, enhancing infiltration and eventual water use by evapotranspiration (Eagleson 1978), meaning more water is retained in soils and less likely to contribute to stream runoff. Increases in LAI, as we saw with poplar establishment, can reflect locally greater water availability (Nemani and Running 1989) because plant water use tends to scale with LAI variations (Hoek van Dijke et al. 2020; Lindroth et al. 2008; Duursma et al. 2009), as has been observed among other sub‐arctic woodlands (Launiainen et al. 2016). Precipitation interception losses also increase with LAI (Kang et al. 2018). A cursory calculation of potential evapotranspiration (Hamon 1963) and 1991–2020 climate normals from the nearby Wonder Lake Climate Station (Denali N‐27; Alaska Climate Research Center 2024) shows that potential evapotranspiration exceeds precipitation in the early growing season (May and June, with a 142 mm of potential evapotranspiration relative to 122 mm of precipitation). Thus, drawdowns in soil moisture should occur then, and greater subsurface storages reduce the likelihood of transpiration being down‐regulated due to plant water shortages. Thus, increases in poplar cover and functional soil depth should lead to smaller fractions of incoming precipitation, especially in summer, becoming streamflow.

Changes associated with poplar invasions also have significant implications for carbon budgets. Higher LAI implies more light interception and more potential gas exchange, which together lead to higher productivity (Lindroth et al. 2008; Duursma et al. 2009; Lagergren et al. 2008). These LAI differences are especially apparent in Terraces 3 and 4, where LAI is ~44% higher in the poplar plots. This higher LAI is associated with substantial accumulation of tree basal area (Table 1), representing a considerable aboveground carbon stock in stem biomass. Using the basal area and height data (Table 1) in a general biomass model developed for northern tree species (Lang et al. 2016) leads to estimates of 18.5, 13.8, and 36.7 Mg/ha of biomass in the poplar trees of the Terrace 2, 3, and 4 poplar plots, respectively; scaling these values to carbon stock density, assuming 50% of biomass is carbon (Lamlom and Savidge 2003), yields 9.2, 6.9, and 18.3 Mg C/ha, respectively. A carbon stock assessment of Alaska reported 5.6 and 18.1 Mg C/ha in aboveground carbon stocks for tussock tundra and shrub tundra ecosystems (Genet et al. 2016). Given that the poplar ecosystem also includes the shrub understory (taller than the shrubs in non‐poplar plots), the poplars in Terrace 4 can result in a doubling of aboveground biomass, which constitutes a ~6‐fold increase relative to estimates for the tundra ecosystem that occupies Terrace 5. This, however, is in stark contrast to what could be expected of belowground carbon stocks, as the same study reports Alaska deciduous forest to have soil carbon stocks of 239 Mg C/ha, much lower than reported values of 625 Mg C/ha for tussock tundra ecosystems (Genet et al. 2016). Warming soils can yield major respiration fluxes in boreal systems (Crowther et al. 2016), meaning shifts away from permafrost soils could imply loss of a major carbon sink (Hugelius et al. 2014; Schuur et al. 2022; Turetsky et al. 2012; Fisher et al. 2016). Further research should seek to understand how the re‐engineering of these ecosystems with balsam poplar invasion could drive broader landscape‐scale changes in how carbon and water are stored and cycled.

## 6 Conclusion

Widespread changes in vegetation communities have been documented concurrent with significant warming across the Circumpolar North in recent decades. However, air temperature changes alone often fail to drive treeline or range expansion of forest species without associated shifts in ecosystem processes that create favorable conditions for vegetation growth. The vegetation mosaic at this study site provides a natural experiment; if the landscape had undergone a complete transformation, it would be unclear whether a feedback relationship was at play or if the climate change alone was driving the shift. This paper tests hypotheses regarding interactions among snow depth, soil characteristics, and vegetation structure that have been previously discussed as having a role in landscape and ecosystem succession (Viereck 1966; Roland et al. 2016). Our results support the hypothesis of a feedback cycle in which the taller structure of poplar woodlands harbor deeper snowpacks, enhancing soil insulation and increasing water input—factors that, in turn, promote further poplar establishment. Specifically, we find that poplar woodlands are associated with greater leaf area index (Figures 3 and 5), deeper functional soil layers (Figures 3 and 5), and deeper snowpacks (Figures 4 and 5). These relationships suggest the role of snow as a key insulating factor, reducing permafrost development and reinforcing conditions favorable for poplar expansion. This process may contribute to a broader biome shift, transforming would‐be subalpine shrub tundra into a poplar‐dominated forest over time (which often serves as a successional bridge to spruce forest). However, the long‐term trajectory of this transition remains uncertain. It is unclear whether these shifts will continue uniformly across the landscape or result in a patchwork of different successional outcomes. Moving forward, a more comprehensive understanding of water availability and vegetation water use, particularly by poplar, will be essential for evaluating the hydrologic impacts of this feedback. Further, deeper insights into soil thermal dynamics and hydraulic properties across the study area will be key to explaining patterns of permafrost formation and persistence, or lack thereof. Understanding these coupled dynamics will be critical for predicting future landscape changes in response to ongoing climate shifts.

## Author Contributions

**Johanne O. Albrigtsen:** conceptualization (equal), data curation (lead), formal analysis (lead), funding acquisition (equal), investigation (lead), methodology (equal), project administration (equal), supervision (equal), visualization (lead), writing – original draft (lead), writing – review and editing (lead). **Sarah E. Stehn:** conceptualization (equal), data curation (equal), funding acquisition (lead), investigation (equal), methodology (equal), project administration (equal), resources (equal), writing – review and editing (supporting). **Carl Roland:** conceptualization (supporting), data curation (supporting), writing – review and editing (supporting). **Scott T. Allen:** funding acquisition (equal), methodology (equal), project administration (equal), resources (equal), supervision (equal), writing – original draft (equal), writing – review and editing (equal).

## Conflicts of Interest

The authors declare no conflicts of interest.

## Data Availability

The data supporting the findings of this study are openly available in Zenodo at https://doi.org/10.5281/zenodo.15263437 (Albrigtsen 2025).
